# Neurological manifestations of Ehlers-Danlos syndrome(s): A review

**Published:** 2014-10-06

**Authors:** Marco Castori, Nicol C. Voermans

**Affiliations:** 1 Department of Medical Genetics, San Camillo-Forlanini Hospital, Sapienza University of Rome and San Camillo – Forlanin, Rome, Italy; 2 Department of Neurology, Radboud University Medical Centre, Nijmegen, Netherlands

**Keywords:** Brain, Developmental Delay, Ehlers-Danlos Syndrome, Fatigue, Headache, Neuromuscular

## Abstract

The term “Ehlers-Danlos syndrome” (EDS) groups together an increasing number of heritable connective tissue disorders mainly featuring joint hypermobility and related complications, dermal dysplasia with abnormal skin texture and repair, and variable range of the hollow organ and vascular dysfunctions. Although the nervous system is not considered a primary target of the underlying molecular defect, recently, increasing attention has been posed on neurological manifestations of EDSs, such as musculoskeletal pain, fatigue, headache, muscle weakness and paresthesias. Here, a comprehensive overview of neurological findings of these conditions is presented primarily intended for the clinical neurologist. Features are organized under various subheadings, including pain, fatigue, headache, stroke and cerebrovascular disease, brain and spine structural anomalies, epilepsy, muscular findings, neuropathy and developmental features. The emerging picture defines a wide spectrum of neurological manifestations that are unexpectedly common and potentially disabling. Their evaluation and correct interpretation by the clinical neurologist is crucial for avoiding superfluous investigations, wrong therapies, and inappropriate referral. A set of basic tools for patient’s recognition is offered for raising awareness among neurologists on this underdiagnosed group of hereditary disorders.

## Introduction

**Figure 1:**
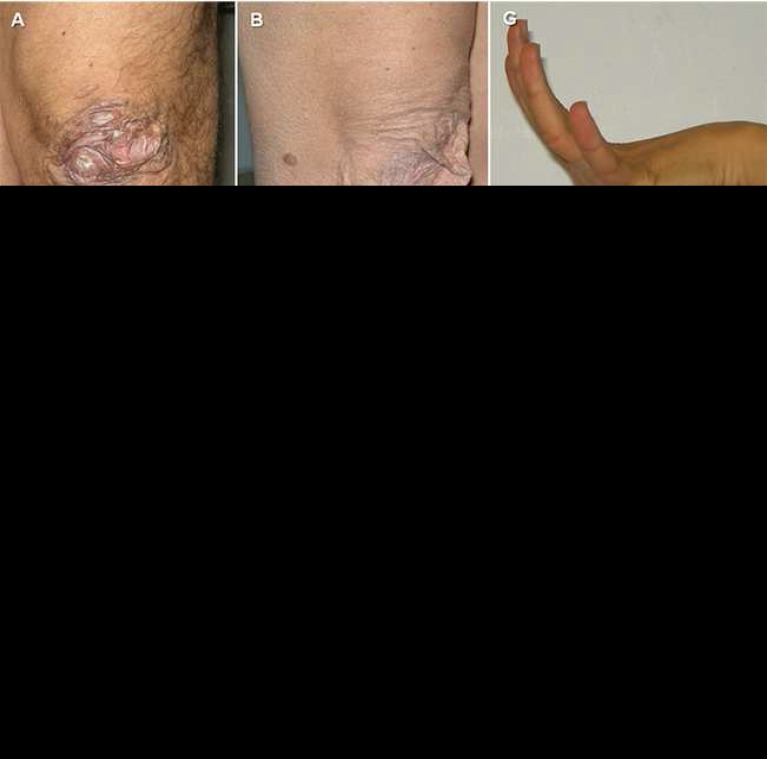
Examples of cutaneous and articular manifestations of Ehlers-Danlos syndrome (EDS). Papyraceous scars at knees in a adult man (A) and woman (B) with classic EDS. Various degrees of skin hyperextensibility (C, D). Extensive easy bruising due to capillary fragility in a adult (E) and child (F). Joint hypermobility at fingers (G). Genu recurvatum (H)

Since the first description in the medical literature,^1^^,^^2^ the definition of Ehlers-Danlos syndrome (EDS) is significantly changed and actually refers to an expanding spectrum of heritable connective tissue disorders sharing the variable triad of (i) generalized joint hypermobility and related osteoarticular complications, (ii) dermal dysplasia extending from minor changes of skin texture to clinically relevant skin fragility and defective scarring, and (iii) vascular and internal organ fragility with proneness to traumatic injuries and spontaneous ruptures, dissections and prolapses (Figure 1). Nosology of EDS mirrors its heterogeneous clinical manifestations and, at present, comprises six major forms or variants (i.e., classic, hypermobility, vascular, kyphoscoliotic, arthrochalasis and dermatosparaxis types)^3^ (Table 1), as well as a growing list of apparently rarer subtypes (Table 2). At present, the clinical suspect of many EDS variants, can be confirmed by specific laboratory investigations, particularly molecular testing.^4^ Nevertheless, the EDS hypermobility type (EDS-HT), which is one of the most common variants, is still a clinical diagnosis based on subtle and likely unspecific features, and shows a nearly complete phenotypic overlap with the joint hypermobility syndrome (JHS) (Table 3), a heritable rheumatologic condition defined by the Brighton criteria.^5^^-^^7^ The question whether EDS-HT and JHS are also identical at the molecular level is still a matter of debate.^8^

This implies that while a group of patients receive a stringent laboratory confirmation of rarer, but well-defined EDS variants, all others, who fall within the blurring spectrum of JHS/EDS-HT, often escape practitioner’s attention and sometimes develop disabling, but potentially preventable complications. In line with this, the presumed cumulative frequency of 1 in 5000 individuals in the general population for EDS^9^ is likely an underestimation and the real prevalence of a single more common variants may be much higher as recently proposed for JHS.^10^

Recurrent muscle cramps have been reported in EDS by Beighton and Horan since the late sixties.^11^ After this early paper, which actually focused on orthopedic aspects of EDS, many other reports (reviewed in the following sections) have highlighted a possibly prominent involvement of the nervous system in this condition. Nevertheless, most works describe single patients or small case series, contributing to the lack of awareness of neurological manifestations of EDS. In 2009, Voermans et al., for the first time, illustrate their findings in 40 patients with various forms of EDS and demonstrate a subtle, but significant neuromuscular involvement in this condition.^12^

**Table 1:** Villefranche criteria for the six major subtypes of Ehlers-Danlos syndrome

**Common variant**	**Inheritance**	**Causative gene(s)**	**Major criteria**	**Minor criteria**
Classic	AD	COL5A1, COL5A2	Skin hyperextensibility Widened atrophic scars Joint hypermobility	Smooth, velvety skin Molluscoid pseudotumors Subcutaneous spheroids Complications of joint hypermobility Muscle hypotonia, motor delay Easy bruising Manifestations of tissue extensibility and fragility Surgical complications Positive family history
Hypermobility	AD	Mostly unknown	Hyperextensible and/or Smooth, velvety skin Generalized joint hypermobility	Recurring joint dislocations Chronic joint/limb pain Positive family history
Vascular	AD	COL3A1	Thin, translucent skin Arterial/intestinal/uterine fragility or rupture Extensive bruising Characteristic facial appearance	Acrogeria Hypermobility of small joints Tendon and muscle rupture Talipes equinovarus Early-onset varicose veins Arteriovenous, carotid-cavernous sinus fistula Pneumothorax/pneumohemothorax Gingival recessions Positive family history, sudden death in a close relative
Kyphoscoliotic	AR	PLOD1	Generalized joint hypermobility Congenital hypotonia Congenital and progressive scoliosis Scleral fragility and rupture of the ocular globe	Tissue fragility, including atrophic scars Easy bruising Arterial rupture Marfanoid habitus Microcornea Osteopenia/porosis Positive family history
Arthrochalasis	AD	COL1A1, COL1A2	Generalized joint hypermobility with recurrent subluxations Congenital bilateral hip dislocation	Skin hyperextensibility Tissue fragility, including atrophic scars Easy bruising Hypotonia Kyphoscoliosis Osteopenia/porosis
Dermatosparaxis	AR	ADAMTS2	Severe skin fragility Sagging, redundant skin	Soft, doughy skin texture Easy bruising Premature rupture of fetal membranes Large hernias (umbilical, inguinal)

AD: Autosomal dominant; AR: Autosomal recessive; EDS: Ehlers-Danlos syndrome. No clear indication for using these criteria in the establishment of a firm clinical suspect of a specific EDS subtype is specified. However, the presence of at least 1 major and 1 minor criteria is usually necessary for proceeding in molecular confirmation of EDS subtypes with a known, prevalent molecular cause. The presence of at least two major criteria is strongly indicative for a definite diagnosis of the specific EDS subtype. Adapted from Beighton et al.^3^

**Table 2:** Rare variants of Ehlers-Danlos syndrome with currently unavailable clinical diagnostic criteria

**Rare variant**		**Inheritance**	**Gene(s)**
Association with 21 α-hydroxylase deficiency		AR	TNXB, CYP21B
Association with Gilles de la Tourette syndrome		AD	HDC
Association with parodontitis		AD	Unknown
Association with periventricular heterotopia		XLD	FLNA
Brittle cornea syndrome type 1		AR	ZNF469
Brittle cornea syndrome type 2		AR	PRDM5
Cardiac-valvular		AR	COL1A2
Classic with arterial rupture		AD	COL1A1
Ehlers-Danlos syndrome/osteogenesis imperfecta overlap		AD	COL1A1, COL1A2
Ehlers-Danlos syndrome-like due to 6q27 deletion	Sporadic/AD		Not applicable
Kyphoscoliotic with myopathy and deafness		AR	FKBP14
Musculocontractural, type 1		AR	CHST14
Musculocontractural, type 2		AR	DSE
Overlap phenotype due to COL3A1/COL5A2/MSTN haploinsufficiency		AD	COL3A1, COL5A2, MSTN (deletion)
Progeroid		AR	B4GALT7
Spondylocheirodysplasia		AR	SLC39A13
Tenascin X-deficient		AR, AD (?)	TNXB

AD: Autosomal dominant; AR: Autosomal recessive; XLD: X-linked dominant; EDS: Ehlers-Danlos syndrome

**Table 3:** Brighton criteria for the joint hypermobility syndrome

**Criteria**
Major criteria
Beighton score ≥ 4/9
Arthralgia for > 3 months in > 4 joints
Minor criteria
Beighton score 1-3
Arthralgia in 1-3 joints
History of joint dislocations
Soft tissue lesions > 3
Marfan-like habitus
Skin striae, hyperextensibility, or scarring
Downslanting palpebral fissures, lid laxity, myopia
History of varicose veins, hernia, visceral prolapse
Agreement: Both major, or 1 major and 2 minor, or 4 minor criteria. Criteria major 1 and minor 1 are mutually exclusive as are major 2 and minor 2.
Source: Grahame et al. and subsequent modifications (see, for example, Tinkle et al.)

A recent review, primarily intended for a readership of child neurologists, emphasizes a heterogeneous and potentially disabling neurological involvement in EDS with a wide range of apparently low-frequency, but possibly subtype-specific anomalies of the nervous system.^13^ In addition, at least for JHS/EDS-HT, musculoskeletal pain, fatigue and headache represent the master contributors to patients’ referral to the practitioner and quality of life deterioration.^14^ Now, it is clear enough that the understanding of the extent of nervous system involvement and of its underlying pathogenic processes represents a nodal point for better assessing EDS patients and developing more efficient rehabilitation and prevention strategies.

In this paper, we review pertinent literature reporting a seemingly increased rate of neurological features in EDS. Our aim was to offer a practical reference to the clinical neurologist in considering this condition in subjects with an apparently primary neurological condition, as well as to those taking part to the multidisciplinary team involved in the management of EDS patients.

**Search Methods**

This work was intended as a narrative review summarizing actual evidence on nervous system involvement in EDS. A PubMed search was carried out matching the terms “EDS” OR “hypermobility syndrome” with the following: central nervous system, brain, myelum, spine, stroke, epilepsy, muscle, myopathy, peripheral nerve(s), polyneuropathy, (peripheral) nervous system, and nerve. Papers were selected when reporting EDS/JHS patients with neurological manifestations. The reference list of selected papers was further searched for additional reports. Our findings were reported in the following paragraphs.

**Pain**

Pain is now accepted as a common and potentially disabling feature of various forms of EDS, especially JHS/EDS-HT.^14^ This is testified by the inclusion of chronic musculoskeletal pain as a major item in the Brighton criteria for JHS^5^ and minor feature in the Villefranche criteria for selected EDS subtypes.^3^ Conversely, in the early EDS literature, pain was recognized as a marginal finding, usually occurring acutely due to (sub) luxations, spontaneous rupture of vessels^15^ and internal organs,^16^ or recurring in relation to piezogenic pedal papules^17^ or gynecologic/obstetric complications^18^ In 1994, Lumley et al. first pointed out a possible role of chronic pain in the quality of life of EDS patients by studying psychosocial functioning in 48 subjects with various forms of EDS.^19^ Furthermore, the repeated evidence of resistance to local anesthetics in both EDS-HT^20^ and JHS^21^ suggests a more complex effect on pain perception and modulation processes in EDS, since the very end of the last century. Scientific awareness on the clinical significance of chronic pain in EDS increased in 2010, when Voermans et al. published the results of their questionnaire study on 273 patients with various forms of EDS (mainly, classic, hypermobility and vascular types) and found pain as common, severe, and associated with functional impairment. Interestingly, pain seemed more severe in EDS-HT than classic and vascular types, and pain severity was correlated with hypermobility, dislocations and previous surgery.^22^ This preliminary observation prompted the same research group to state that pain and fatigue are possible important determinants of disability of EDS-HT.^23^ On a clinical perspective, chronic-recurrent joint pain is the most common presentation of pain in JHS/EDS-HT, which is reflected by the applicable diagnostic criteria.^3^^,^^5^ Additional manifestations of musculoskeletal pain in JHS/EDS-HT and perhaps other EDS subtypes include muscle cramps,^11^ fibromyalgia^24^^,^^25^ and compression/peripheral neuropathies.^26^^,^^27^ Pain is not limited to the musculoskeletal system and often presents with visceral involvement, in the form of various gastrointestinal chronic and recurrent symptoms,^28^ as well as dyspareunia, vulvodinia and dysmenorrhea.^29^^,^^30^ Headache is also common in EDS^31^ and is discussed in a separate section. The study of the natural history of JHS/EDS-HT tells us that pain is a changing feature in EDS and its functional consequences are influenced by the adaptation strategies that the various structures of the affected individual develop, as a whole, to face repetitive pain perception.^14^ Accordingly and following previous postulations,^14^ a recent work by Rombaut et al. demonstrates the presence of hyperalgesia in EDS-HT.^32^ This finding may represent a preliminary proof for the action of central sensitization processes in those EDS patients who develop the most severe disabilities associated to pain.

**Fatigue**

Though largely ignored in the past, severe fatigue is now considered a common accompanying feature of EDS, particularly EDS-HT, as it is reported in up to 84% of the patients.^33^ Similar results are obtained by other research groups.^34^^,^^35^ More specifically, the frequency (and, perhaps, severity) of fatigue is influenced by age with a rate of 28% in the first decade of life to 90% in adults over 40 years of age.^35^ In JHS/EDS-HT, the impact of fatigue on daily life is often equal or more dramatic than the impact of pain;^33^ a fact that underscores the importance of fatigue for both assessment and treatment planning in these patients. A complex presentation of fatigue resembling chronic fatigue syndrome according to Fukuda et al.^36^ is reported in most adults with JHS/EDS-HT.^37^

Some possible contributors to fatigue-related disability have been investigated in EDS and include sleep disturbances, concentration problems, social functioning, self-efficacy concerning fatigue, and pain severity.^33^ A few experimental studies demonstrate that fatigue associates with muscle weakness,^38^^,^^39^ worsens with exercise^40^ and affects gait pattern.^41^ Recently, dysautonomia was recognized as one of the most relevant pathogenic factors influencing fatigue onset and evolution. This feature, mostly studied in JHS/EDS-HT, often presents with orthostatic tachycardia syndrome^42^ and/or orthostatic intolerance, and seems to relate to increased sympathetic activity at rest and reduced sympathetic reactivity to stimuli.^43^

**Headache and Head Pain**

In 1996, Spranger et al. first report headache, drop attacks and white matter abnormalities in a 37-year-old woman with EDS with parodontitis.^44^ 1 year later, a larger study on 51 individuals with different forms of EDS (including 13 patients with classic type, 28 with EDS-HT, 1 with JHS, 7 with vascular type, and 2 with unclassified type) showed that neck pain and headache is present in 30-40% of cases.^31^ A subsequent case series reports nine EDS patients presenting with various forms of headache, including (i) migraine with aura, (ii) migraine without aura, (iii) tension-type headache, (iv) a combination of tension-type headache and migraine, and (v) post-traumatic headache.^45^ Additional works on small case series confirm the high prevalence of headache in JHS/EDS-HT.^34^^,^^46^ More specifically, Bendik et al. show that migraine (with or without aura) is approximately three times more common among a group of 28 women with JHS/EDS-HT compared with 232 controls with a cumulative frequency of 75% (3/4).^47^ In a 36-year-old woman with orthostatic headache and idiopathic intracranial hypertension, the diagnosis of JHS was reached 2 years later the onset of head pain.^48^ This patient displayed increased IGF-1 plasma levels and the authors speculated on a possible pathogenic link. Head pain is not limited to headache in EDS. In a cohort of 31 EDS patients (including 16 with EDS-HT, nine with classic EDS and six with vascular EDS), De Coster et al. demonstrated temporomandibular joint dysfunction in 100% of the cases, unilateral myofascial pain (i.e., temple headache) in 83%, and unilateral and bilateral temporomoandibular joint pain in 28% and 51% of the patients, respectively.^49^

While many EDS patients may develop chronic or recurrent headache, individuals with EDS types with vascular fragility (e.g., vascular EDS, classic EDS with arterial rupture and kyphoscoliotic EDS) are at increased risk of sudden headache secondary to acute cerebrovascular disease. In particular, acute headache may occur together with ophthalmoplegia or tinnitus due to spontaneous, direct caveronous-carotid fistulain this EDS type.^50^^,^^51^ Additional observations point out possible associations between localized or generalized joint hypermobility and specific subsets of primary and secondary types of headache, including new daily persistent headache^52^ and headache attributed to spontaneous (idiopathic) cerebrospinal fluid leakage.^53^^,^^54^ Cervical spine hypermobility/dysfunction is also anecdotally considered a predisposing factor for cervicogenic headache^55^^,^^56^ and neck-tongue syndrome.^57^^-^^59^ In line with this, Di Palma and Cronin report a 27-year-old woman with classic EDS with a long-lasting pulsating headache associated with C2 dislocation.^60^

**Stroke and Cerebrovascular Disease**

Most reports on stroke and cerebrovascular disease in EDS concern vascular EDS and include intracranial aneurysms, subarachnoid hemorrhage, spontaneous arterial dissection and cavernous sinus fistula. In patients with classic EDS, aneurysms occasionally occur, whereas other vascular abnormalities are rare. In fact, the presence of stroke in patients with cutaneous features of classic EDS should prompt to molecular investigation for the classic variant with arterial rupture due to mutations in COL1A1.

In 1990, Schievink et al. reported two vascular EDS patients, one with a spontaneous internal carotid artery dissection and one with an aneurysmal subarachnoid hemorrhage and multiple aortic dissections.^61^ Both patients were deficient in collagen Type III, analyzed in cultured skin fibroblasts. Subsequently, the same author reported a 20-year-old woman with vascular EDS, who presented with a spontaneous carotid-cavernous fistulae and a cervical artery dissection.^62^ The clinical features of 16 previously published cases of spontaneous carotid-cavernous fistulae were also reviewed.^62^ Since collagen Type III deficiency plays a role in the pathogenesis of intracranial saccular aneurysms, it is likely to be involved in the pathogenesis of carotid cavernous fistulas and dissections of the cervical arteries.^61^ In 1995, North et al. studied the cerebrovascular complications in a cohort of 202 patients with vascular EDS.^63^ 19 patients had cerebrovascular complications, including intracranial aneurysms with secondary hemorrhage, spontaneous carotid-cavernous sinus fistula and cervical artery dissection. The aneurysms typically develop in the cavernous sinus or directly adjacent to it, and bilateral and recurrent carotid aneurysms have also been reported.^64^^-^^68^ Aneurysmal rupture can occur spontaneously or during vigorous activity.^61^^,^^63^ A carotid-cavernous fistula can develop after minor head trauma, but most occur spontaneously, in many cases after a ruptured internal carotid artery aneurysm within the cavernous sinus.^62^ These carotid-cavernous fistula often present with exophthalmos, chemosis, pain, opthalmoplegia and bruits, which result from their high blood flow which allows pressurized arterial blood to connect directly to the cavernous sinus resulting in venous hypertension and reversal of venous drainage.^69^

Although uncommon, vascular EDS is a potential cause of stroke in young people.^70^^-^^72^ In typical cases, the disorder is readily identifiable by clinical inspection and targeted molecular testing. However, the phenotypic variability of vascular EDS could lead to under diagnosis, especially in an emergency setting.^71^ Hence, a full clinical history and examination and a complete familial history should be emphasized as often only the combination of all these data will raise suspicion of vascular EDS. This is crucial since the diagnosis has important implications for acute and long-term management and, potentially, for other family members.^71^

In vascular EDS, the traditional approach has been to treat such complications conservatively unless they are life-threatening. Recent reports challenge this treatment paradigm.^73^ In a randomized study, treatment with the beta-blocker Celiprolol was shown to be associated with a three-fold decrease in arterial rupture in vascular EDS.^74^ Novel approaches using endovascular therapy with coil embolization have shown good results in the treatment of ruptured pseudoaneurysms and carotid-cavernous fistulas.^69^^,^^73^ Nevertheless, complications are frequently reported. Anticoagulation therapy may result in increased bruising or bleeding and should be used with caution.^63^^,^^75^ Because conventional angiography may exacerbate severe complications, non-invasive magnetic resonance angiography is the investigation of choice.

**Brain and Spine Structural Anomalies**

**Figure 2:**
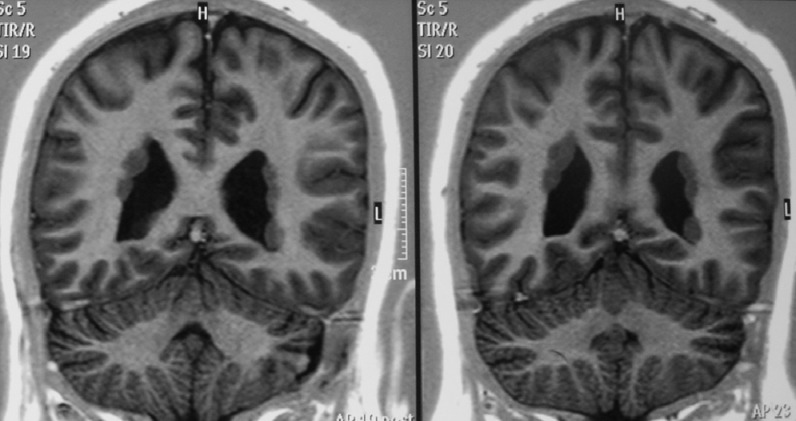
Periventricular heterotopias in a 37-year-old woman with features of classic Ehlers-Danlos syndrome

Brain magnetic resonance imaging (MRI) is usually unremarkable for major structural anomalies in EDS. Therefore, with the exception of acute presentations with the suspect of cerebrovascular disease, such an investigation usually does not take place in the baseline assessment of EDS patients. Nevertheless, the presence of clear-cut X-linked dominant transmission and/or the coexistence of seizures should elicit the exclusion of the rare form of EDS with periventricular heterotopias (Figure 2) often due to mutations in FLNA.^76^^,^^77^ Single reports also associate EDS with polymycrogyria,^78^ agenesis of the corpus callosum,^79^ dilatation of the 4^th^ ventricle, supracerebellar cistern and lateral ventricle, or disproportional enlargement of anterior horn of lateral ventricle,^80^ but the consistency of such associations awaits confirmation. Conversely, clinical practice suggests a high rate of degenerative findings, in the form of generalized, early-onset spondylosis and disc hernias, at spine MRI. However, this evidence remains anecdotal^81^ and needs systematic confirmation. Spine MRI may also show meningeal cysts as reported in a 7-year-old girl with kyphoscoliotic EDS,^82^ in an adult with classic EDS^83^ and, with a low frequency, in JHS/EDS-HT.^84^ In EDS, meningeal (or Tarlov) cysts are apparently rare and very few or single. The 40-year-old woman with multiple and bilateral Tarlov cysts along the entire spine and originally defined an example of (unclassified) EDS by Isono et al.,^85^ can be best labeled with the diagnosis of lateral meningocele syndrome,^86^ an apparently distinct form of hereditary connective tissue disorder with severe thecal involvement.^87^ The clinical significance of meningeal cysts is still unknown in EDS, although a link with orthostatic headache due to spontaneous cerebrospinal fluid leaks may be inferred,^88^^,^^89^ similarly as in Marfan syndrome. The concurrence of Chiari malformation (Type I) is a further apparently underreported feature of EDS, as to date observed in single patients only.^45^^,^^46^ Nevertheless, in a surgically-oriented paper on hundreds of patients originally ascertained for symptomatic Chiari malformation, a subgroup of them, showing a high rate of recurrence after surgery and multiple features of an underlying connective tissue disorder, presented minor, but measurable neuroradiologic features of occipitoatlantoaxial instability: posterior gliding of the occipital condyles, and reduction of the clivus-axis angle, clivus-atlas angle, and atlas-axis angle in the upright position.^90^ In line with this, an early report describes two vascular EDS patients with radiologically evident atlantoaxial subluxation.^91^ Hence, investigating for cerebellar tonsils herniation and an occult occipitoatlantoaxial instability is relevant during the assessment of patients with occipital or postural/orthostatic headache, and/or additional unexplained neurological symptoms, such as upper limb paresthesias and weakness.

**Epilepsy**

The mechanisms explaining the possible association between EDS and seizures are likely heterogeneous. In 1981, Cupo et al. reported a 30-year-old woman with EDS who died due to an intractable ventricular fibrillation due to myocardial infarction. She also presented seizures, aneurysms of the sinus of valsalva and severe panacinar emphysema. Necropsy finding included cerebral heterotopias.^92^ Since this early description, additional reports suggest the existence of a distinct form of EDS with cutaneous features resembling the classic type, coupled with periventricular subependymal heterotopias.^76^ In most cases, this presentation is transmitted in an X-linked dominant pattern and is mutated in the Filamin A.^77^^,^^93^ Furthermore, Jacome reports seven EDS patients, two of them affected by occipital-horn disease which is actually moved outside the EDS classification, with epilepsy and various concomitant neurological complications.^94^ Of the five remaining individuals with more stringent diagnosis of EDS, possible nervous system determinants for seizures include basilar artery hypoplasia, hemispheric atrophy, venous parietal angioma, previous intracranial bleed and previous stroke. Additional reports of EDS with seizures include a 29-year-old man with unclassified EDS and bilateral frontocentral and frontoposterior polymicrogyria with hypoplasia of the cerebellar vermis, a 20-year-old woman with EDS-HT and bilateral perisylvian polymicrogyria,^78^ and a 16-year-old man with classic EDS and agenesis of the corpus callosum.^79^ These reports suggest a likely increased rate of epilepsy in EDS. Although in many cases structural brain anomalies (particularly, periventricular heterotopias) may be identified, mechanisms linking seizures to a hereditary defect of the connective tissue remain poorly studied.

**Muscular Findings**

EDS is associated with a variety of neuromuscular features, and vice versa, a variety of congenital myopathies display joint hypermobility and, to a lesser extent skin changes.^95^ Awareness of this clinical overlap between inherited connective tissue disorders and certain congenital myopathies might be helpful in recognition of these rare disorders.^96^ The initial reports of Beighton already included a high prevalence of myalgias and nocturnal muscle cramps involving the calves, especially in EDS-HT.^11^^,^^97^ This author also detected hypotonia, progressive muscle weakness, poorly developed musculature, and scapular winging, all without signs of concomitant myopathy. It was suggested that the muscle hypotonia might have a basis in the abnormality of the collagen in muscle sheaths rather than in the muscle fibers itself, but this was deemed unlikely. In contrast, these symptoms were considered to be secondary to avoidance of exercise because of the hypermobility and instability of joints.

This might have contributed to the relative neglect of muscle features in EDS, and for years only few case reports pointed to these EDS manifestations. Banerjee et al. reported a 9-year-old boy presented with delayed walking and abnormal gait.^98^ The presence of marked muscle hypoplasia with hypotonia led to the initial diagnosis of primary muscle disease; later, he was found to have hyper-extensible and fragile skin and hyper mobile joints, and EDS was diagnosed. The second case was reported by Bertin et al. showing combination of EDS and muscular dystrophy in a 16-year-old male patient.^99^ Furthermore, Palmeri et al. reported the association of chronic muscle pain and cramps and the vascular type of EDS.^100^ The first study on the physiological basis of muscle weakness in EDS was performed by Bilkey et al. demonstrating that muscle weakness was primarily due to the alterations in the connective tissue surrounding the muscle cells.^101^

In 2009, Voermans et al. performed a prospective study in 40 genetically or biochemically confirmed patients with various forms of EDS (vascular EDS, classic EDS, EDS-HT and TNXB-deficient EDS), showing that mild-to-moderate neuromuscular involvement is common in various types of EDS.^12^ Patients reported muscle weakness, myalgia, easy fatigability, and limited walking distance. Physical examination revealed mild to moderate muscle weakness, reduction of vibration sense, and mild impairment of mobility and daily activities. Ancillary investigations showed only mild a specific myopathic signs. The findings of this study have increased awareness of neuromuscular symptoms in EDS patients. Muscle biopsies to exclude the presence of a co-existent myopathy might not be necessary if typical clinical and neurophysiological findings are encountered and the diagnosis is confirmed by an expert in the field.

Another finding in this study was the remarkable relation between residual TNX levels and degree of neuromuscular involvement, compatible with a dose-effect relation. This points to the role of the extracellular matrix defect in muscle and peripheral nerve dysfunction in EDS^12^ This was confirmed in physiological studies in 

**Neuropathy**

Brachial and/or lumbosacral plexus neuropathies and compression mononeuropathies have been reported in several types of EDS.^26^^,^^103^^-^^106^ Although the study of Voermans et al. focused primarily on muscle, signs of peripheral nerve involvement in EDS were also observed. Nerve conduction studies demonstrated axonal polyneuropathy in 13% of patients, mainly of the vascular and TNXB-deficient types.^12^ Recently, a study on entrapment neuropathies and polyneuropathies in EDS-HT showed a higher prevalence of neuropathic symptoms (paresthesias/numbness in hands and/or feet) than of neurophysiological and ultrasound evidences of focal or diffuse nerve involvement. This might be due to presence of radiculopathy or small fiber neuropathy, which was not addressed in this study. Furthermore, a high prevalence of ulnar nerve subluxation/luxation at the elbow was detected on dynamic ultrasound.^107^

The pathophysiological mechanism of peripheral neuropathy in EDS-HT seems related to the dislocations and subluxations due to ligament and capsular laxity which cause abnormal stretching of or pressure on peripheral nerves, and, thus can result in neuropathy or plexopathy. Increased vulnerability of peripheral nerves to stretching or pressure directly linked to the underlying genetic defect might also be involved. TNXB or Collagen I, III or V deficient epi-, peri- and endoneurium - the connective tissue of peripheral nerves might fail to resist excessive mechanical stress.^26^

**Developmental Features**

Global developmental delay evolving in intellectual disability is rare in EDS. Given the high frequency of joint laxity in the general population as well as in disabling genetic conditions, the presence of a true global developmental delay should prompt the practitioner to search for different diagnoses other than EDS. The possible exception to this rule of thumb is represented by the EDS-like phenotype associated with 6q27 chromosome deletions,^108^ which may be detected by CGH-array, a first-line investigation for EDS-like subjects with intellectual disability. Conversely, relatively robust data support a tight link between joint hypermobility and reduced motor performance in children. In fact, while early work fails to detect an association between joint hypermobility and neurodevelopmental attributes,^109^ two more recent studies demonstrate that joint hypermobility is more common among children with developmental coordination disorders.^110^^-^^112^ Complementarily, abnormal gait, clumsiness and poor coordination are common findings in children with a previous diagnosis of JHS/EDS-HT.^113^^,^^114^ Such an apparently selective influence of congenital joint hypermobility on coordination could be the consequence of an impairment of proprioception in critical phases of motor development. This hypothesis lies on the repeated evidence of defective proprioception in JHS/EDS-HT.^115^^-^^119^ The combination of generalized JHM and the above-mentioned motor attributes of JHS/EDS-HT children explain why they perform well at sports, such as gymnastics, ballet and dancing, and often improve their motor outcome by exercise. The natural propensity to specific sports, which contrasts the poor coordination skills reported by many patients, is a primary feature of the “first phase” of disease progression recently outlined for JHS/EDS-HT.^14^^,^^35^^,^^46^ This neurodevelopmental profile seems extremely common in JHS/EDS-HT and, presumably, in other hereditary connective tissue disorders.

## Conclusion

This overview on neurological manifestations of EDS demonstrates a largely unrecognized set of central and peripheral nervous system features in patients with heritable connective tissue disorders. The familiarity that any neurologist has with some of these manifestations when reported in the general population, such as cerebrovascular disease, headache, myalgia and fatigue, poses neurologists in a privileged position for promptly recognizing EDS. Although the global assessment of EDS patients is, by definition, multisystem and often managed by other specialists, such as rheumatologists and clinical geneticists, the neurologist has a high chance of evaluating still undetected EDS patients with a neurological presentation. In addition, while the pathognomonic features of EDS are not historical heritages of neurology, now, we know that a great proportion of the increased mortality and morbidity of EDS patients is linked to the reverberations that a primary connective tissue derangement has on nervous system development and functions. Hence, all practitioners occasionally or constantly involved in the management of EDS should be better aware of the neurological manifestations of this condition on both clinical and research perspectives.

**Figure 3:**
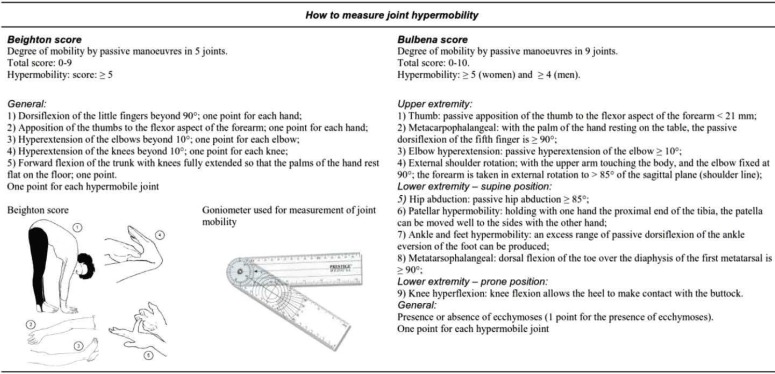
Summary of the Beighton and Bulbena scores for assessing joint hypermobility. The picture of the Beighton score is freely available at: http://www.hypermobility.org/beighton.php

On a practical point of view, the group of EDS types is large with a wide spectrum of manifestations and possible complications. Nevertheless, in this paper reviewing neurological features in EDS, the variability at presentation may be narrowed to a discrete list (Table 4).^120^ Figure 3^120^ illustrates the procedures for assessing generalized JHM by the Beighton score^121^ which is currently included in both Villefranche and Brighton criteria, as well as the alternative Bulbena score, which has proved to be equally efficient in discriminating hypermobile and non-hypermobile subjects.^122^ Both sets have limits and are not ideal in all situations, but may be considered useful first-line screening methods. A positive scoring as illustrated in figure 3 should prompt the clinical neurologist to refer the patient to an expert in the field for diagnosis confirmation and appropriate multidisciplinary management.

**Table 4:** “Red flags” for suspecting Ehlers-Danlos syndromes

**Key clinical clues by category**	
General history	Delayed gross motor milestones Developmental coordination disorder Double-jointed in sports; being proficient at ballet and gymnastics during childhood Showing hypermobility “tricks” during childhood Giving up sports during the teens due to recurrent injuries, pain, and fatigue Mild generalized muscle weakness Unexplained musculoskeletal pain Easy fatigability Paresthesias
Medical history	Recurrent (sub)luxations Tendon and muscle rupture Anal prolapse in childhood Reduced effect of local anesthesia Complications of surgery Easy bruising, sometimes with incorrect suspicion of child abuse Abnormal wound healing with wide atrophic scars Uterus prolapse or cervical insufficiency with premature delivery Early onset varicose veins Pneumothorax or pneumohematothorax Spontaneous arterial rupture (mostly vascular or kyphoscoliotic type) Clubfoot (mostly vascular type) Intestinal or uterine fragility or rupture (mostly vascular type) Severe scoliosis at birth (mostly kyphoscoliotic type) Congenital bilateral hip dislocation (mostly arthrochalasia type)
Family history	Generalized hypermobility/contortionism Sudden death (vascular type)
Physical examination	Generalized joint hypermobility (Beighton score of 5 or more) Increased skin hyperextensibility ( ≥ 3 cm on volar side of lower arm) Smooth, velvety skin Wide, atrophic scars Molluscoid pseudotumors Subcutaneous feroids Thin, translucent skin, characteristic facial appearance, and acrogeria (vascular type) Gingival recessions/(early onset) parodontopathy Microcornea (kyphoscoliotic type) Congenital kyphoscoliosis (kyphoscoliotic or arthrochalasia type)
Neurological examination	Mild proximal weakness (MRC 4) Mild reduction of vibration sense (measured with Rydell-Seiffer tuning fork) Reduction but no absence of tendon reflexes
Ancillary investigations	Nerve conduction studies: reduction of CMAP of distal muscles, mild axonal sensomotor polyneuropathy Electromyography: mixed pattern of both small (myopathic) and larger (neurogenic) units, or predominantly myopathic units CK: normal or mildly elevated (generally < 500) Muscle biopsy: normal or mild myopathic features (increase of fibre diameter variance and internal nuclei) Heart imaging: mitral valve prolapse, or proximal, non-progressive aortic dilatation Bone computer mineralometry: early onset osteopenia

Adapted from Voermans, CMAP: Compound muscle action potential, EDS: Ehlers-Danlos syndrome

## Notes

**How to cite this article: **Castori M, Voermans NC. Neurological manifestations of Ehlers-Danlos syndrome(s): A review. Iran J Neurol 2014; 13(2): 190-208.
